# Self-Management of Chronic Diseases Among Older Korean Adults: An mHealth Training, Protocol, and Feasibility Study

**DOI:** 10.2196/mhealth.9988

**Published:** 2018-06-29

**Authors:** Heejung Kim, Eunhee Park, Sangeun Lee, Mijung Kim, Eun Jeong Park, Soyun Hong

**Affiliations:** ^1^ College of Nursing Yonsei University Seoul Republic Of Korea; ^2^ Mo-Im Kim Nursing Research Institute Yonsei University Seoul Republic Of Korea; ^3^ School of Nursing University at Buffalo Buffalo, NY United States; ^4^ Mapo Senior Welfare Center Seoul Republic Of Korea; ^5^ Seodaemun Senior Welfare Center Seoul Republic Of Korea

**Keywords:** mobile health, feasibility study, chronic disease, eHealth Enhanced Chronic Care Model, elderly, community health service

## Abstract

**Background:**

Most training programs for self-management of chronic diseases in Korea currently involve face-to-face interactions primarily in a health care setting. Therefore, older Koreans living in the community continue to seek other training opportunities for the management of chronic diseases. This has led to the development of new training methods, such as mobile health (mHealth) care, which are valuable in community centers and homes.

**Objective:**

This feasibility study (1) developed an mHealth training protocol to empower community-dwelling elderly individuals to manage their chronic diseases; (2) examined the feasibility of delivering this mHealth training protocol to elderly individuals through mobile tablets and applications (apps); and (3) discussed the contextual and methodological challenges associated with the development of this protocol.

**Methods:**

The mHealth training protocol was developed based on the eHealth Enhanced Chronic Care Model and comprised of four phases. Phase 1 included standardized technology (mobile tablets) training using guidebooks, demonstrations, and guided practice. Phase 2 included provision of standardized information about disease management that was obtained from governmental and professional health care organizations. Phase 3 included provision of training on the use of high-quality mHealth apps that were selected based on individual diagnoses. Phase 4 included encouraging the patients to practice using self-selected mHealth apps based on their individual needs. Quantitative descriptive statistics and qualitative content analyses of user evaluations were used to assess the feasibility and user acceptance of this protocol.

**Results:**

Of the 27 older adults included in this study, 25 completed all 4 weeks of the mHealth training. The attrition rate was 7% (2/27), and the reasons included time conflicts, emotional distress, and/or family discouragement. The men required little or no training for Phase 1, and in comparison with men, women seemed to depend more on the mHealth trainers in Phase 3. Gender, level of education, and previous experience of using smartphones were associated with the speed of learning, level of confidence, and overall competence.

**Conclusions:**

A tailored and personalized approach is required to develop mHealth training protocols for older adults. Self-management of chronic diseases via mHealth training requires careful consideration of the complex nature of human behavior, emotional responses, and familial influences. Therefore, integration of a theoretical, clinical, and technical approach is necessary for the successful development and implementation of an mHealth training program that targets older adults with chronic diseases in a community setting.

## Introduction

Chronic diseases such as cancer, heart disease, diabetes, chronic lower respiratory disease, liver disease, hypertension, and cerebrovascular disease were some of the leading causes of death in Korea in 2014 [1]. According to the Ministry of Health and Welfare report (2014), 90.4% of elderly Koreans exhibited at least one of the chronic diseases listed above, of which 18.2% were diagnosed with one disease, 22.8% with two, and 49.4% with three or more chronic diseases [2]. Statistics Korea (2016) reported that the life expectancy at birth in 2015 was 82.1 years [3], and this differed from the disability-adjusted life expectancy (ie, 70.72 years) by approximately 10 years. This can be attributed to advances in treatment strategies for chronic diseases or long-lasting disabilities for an extended period in late adulthood [4].

Self-management of chronic diseases is essential to minimize their negative influence on the patient’s health status. Therefore, it is imperative to emphasize the importance of active disease management and maintenance of proper health behaviors that promote a better quality of life among older adults [5,6]. To encourage the younger individuals to engage in healthy behaviors at an early stage, it is important that the characteristics of elderly individuals be taken into consideration when designing training protocols to achieve sustainable self-management outcomes [5]. Therefore, there is a considerable need to diversify self-management training protocols to overcome methodological limitations and the effects of aging [7].

However, promoting self-management of chronic diseases among elderly Koreans is challenging. The majority of self-management programs for chronic diseases currently available involve face-to-face interactions through group-based teaching sessions, where general information on diseases is provided by health care providers [7,8]. However, this information-focused approach has not been effective in changing health behaviors [9]. Moreover, most of these programs are only provided for a short period of time (4-12 weeks), and are intensively centered on delivering information about the disease and medication using a one-way communication approach that is directed from the health care providers to the patients [7]. This standardized approach has limitations in that it does not provide a tailored and personalized training experience, despite the fact that most elderly individuals exhibit different levels of progression with regard to their chronic diseases [8,9].

Mobile health (mHealth) training, defined as “medical and public health practice supported by mobile devices, such as mobile phones, patient monitoring devices, personal digital assistants (PDAs), and other wireless devices, [10]” is a newly-developed method of enhancing self-management of diseases [10-12]. It enables users to utilize resources through network services and diverse technological apps in a timely manner and eliminates the need for physical travel [10,12,13]. Furthermore, mHealth services have the potential to empower users to become active to participate in health care practices through interactive communications with health care providers [12]. These mHealth interventions help individuals identify their health care needs, facilitate access to health services, and provide assistance with solving problems related to their illnesses [12,13]. Some studies have reported that mHealth interventions are associated with potential increases in adherence to treatment and disease management, which lead to better patient outcomes across the chronic disease trajectory [10,13].

However, there are significant gaps in the literature focusing on the manner in which mHealth training may assist elderly individuals in self-management of their chronic diseases [10,13-19]. Past studies have emphasized the efficacy of disease-centered training that focuses on a single disease group and is delivered through mobile communication, including telephone calls and short message services, rather than multi-factorial training sessions that integrate apps and Web-based interactive programs [10]. Moreover, there is limited information on how these mHealth training protocols are developed, and whether they have taken key challenges faced by community-dwelling elderly individuals into consideration [10,13,20]. Moreover, there are no theory-based studies that have aimed to develop specific components of mHealth training tailored to elderly Koreans with diverse chronic diseases. Therefore, it is necessary to conduct a feasibility study to evaluate the training protocol prior to conducting any experimental studies [21]. Consequently, we (1) developed an mHealth training protocol to empower community-dwelling elderly individuals to manage their chronic diseases; (2) examined the feasibility of delivering this mHealth training protocol to elderly individuals through mobile tablets and apps; and (3) discussed the contextual and methodological challenges associated with the development of this protocol.

## Methods

### Study Design

This study consisted of two parts: (1) a methodological section that describes the development of the mHealth training protocol, and (2) a descriptive section focusing on evaluation provided by users (N=27) who participated in the mHealth training.

### Developing the mHealth Training Protocol

The eHealth Enhanced Chronic Care Model (eCCM) [22] was used to guide the development of the mHealth training protocol described in this study and to evaluate its feasibility. It explains how adults with chronic diseases become active, educated, engaged, and empowered while working with their health care providers, communities, and social networks in actual and virtual communities along with health care systems [22]. Because the management of chronic diseases in Korean adults is currently centered in health care settings, our mHealth strategies encouraged those living in the community to enhance their integrative knowledge and practice of self-management when dealing with chronic diseases on a daily basis.

The current mHealth training protocol, which focused on the self-management and mHealth training components of the eCCM, included multiple phases: (1) phase 1: standardized technology training; (2) phase 2: provision of general information about chronic diseases; (3) phase 3: mHealth training using pre-selected mobile apps related to individual chronic diseases; and (4) phase 4: active use of self-selected apps based on daily health care needs. Each of these phases has been described below.

Phase 1 used an iPad mini 2 retina (Apple Inc, California, USA) display to provide standardized training to all study participants. This device has several advantages: (1) its size and weight are suitable for carrying around; (2) it has high pixel density in high quality images, which is key for elderly individuals with vision difficulties; and (3) it allows the user to access a diverse range of apps developed internationally [10,11,20,23].

The strategies employed to protect patient anonymity and confidentiality were carefully designed and described to the participants [13,20]. As per the recommendations for ethical conduct of internet, computer, and technology research proposed by the Association of Internet Research [24], a unique code was assigned to each participant for data collection and entry into the database. An anonymous user identification number and password was created by a research assistant, and the participants were provided these to allow them to log into the mHealth device.

Technology training included provision of mobile device guidebooks, standardized demonstrations by the mHealth trainers, guided practice, and 24/7 telephonic trouble-shooting services. After allowing the participants to practice for one week, the mHealth trainers evaluated their proficiency in using the mobile devices independently [20]. In addition, the participants also self-reported their level of confidence in using the mobile tablets [20]. Once the participants were evaluated as being both proficient and confident in using the device, they progressed to Phase 2. However, if participants were not deemed proficient, confident, or comfortable enough with the device, they were asked whether they were willing to continue the training. In case they expressed willingness to continue, additional training was provided by the mHealth trainers a maximum of two more times.

In Phase 2, the mHealth trainers provided information retrieved from the Korean Center for Disease Control [25], Ministry of Health Welfare [26], Korean Academy of Medical Sciences [27], and Korean Diabetes Association [28] on healthy diets, exercise, lifestyle modifications, prevention and management of complications secondary to primary diseases, and medication. This information was retrieved on the basis of the following criteria: (1) the information should focus on a single or multiple chronic disease(s) in a specific individual; (2) the information on the risk factors and consequences of chronic diseases should be evidence-based; (3) the information should be written at a sixth grade or lower level of literacy [29]; and (4) the information should be designed such that it can be efficiently delivered using diverse types of multimedia, including webpage script, video streaming, workbooks, and pictures.

In Phase 3, following assessment of the type of chronic disease, health status, individual needs, and mHealth proficiency of each participant by the mHealth trainers, specific mobile apps including blood pressure measuring tools, fingertip glucose monitoring, tracking log, and interactive games were selected. Only apps that could operate both online and offline were chosen so that users with no internet access could use them freely [10]. Neither the mobile tablet provided nor the apps selected required the participants to enter their personal information. Moreover, all participants were instructed that they were required to log out after using their personal email or other apps on the provided device.

In Phase 4, the feasibility was examined by asking the participants to use the selected apps for at least 30 minutes daily for one week [11]. Participants were free to explore additional apps and download them with the help of the mHealth trainers, if necessary. Upon completion of the mHealth training, equipment was sanitized, inspected for safety, and retested as per the manufacturer’s recommendations and the research protocol before being given to the next participant. Web pages and apps that were not part of the mHealth training program and were used by the participants were not examined in order to protect the participant’s privacy [13,20].

### Feasibility Test

#### Study Participants

A convenience sample of 30 elderly Korean individuals was selected from two community centers. Inclusion criteria were as follows: (1) age ≥60 years; (2) ability to understand Korean; (3) living at home in a community setting; and (4) living with at least one chronic disease, including cardiovascular disease, diabetes mellitus, hypertension, and cerebrovascular disease, which are the most prevalent among elderly Koreans [2]. Those with cognitive impairment, depressive mood, or psychiatric disorders (assessed using the Korean versions of the Mini-Mental Status Examination, MMSE-K [30]; the Geriatric Depression Scale-short form, SGDS-K [31]; and self-reports) were excluded. Following the exclusion of 3 ineligible volunteers, 27 participants commenced with our mHealth training, of whom 25 completed it.

#### Measures

For screening purposes, the MMSE-K and SGDS-K were used to assess cognitive impairments and emotional distress, both of which are barriers to health training. The MMSE is a 30-point scale that is internationally used to assess cognitive function in older adults [32]. The Korean version has been shown to be valid, reliable, and culturally equivalent to the English version, with regard to diagnostic, concurrent, and discriminant validity [30]. The SGDS is a 15-item measure that is used internationally to assess geriatric depression with scores on a range from 0 to 15 [33]. The Korean version has also been shown to be valid, reliable, and culturally equivalent to the English version, in terms of internal consistency and discriminant validity [31]. MMSE scores lower than 24 and SGDS-15 scores higher than 11 indicate significant cognitive impairment and depressive mood, respectively, and these were used as the exclusion criteria for this study [20].

At baseline, all participants completed the questionnaires, including all socio-demographic, clinical, and computer-related questions after screening. Socio-demographic characteristics included age, gender, education, marital status, living status, and employment, whereas the clinical information included the number and types of chronic diseases and comorbidities exhibited, disease duration, and history of medications. Computer-related information included questions on whether the participants had previously used computers, smart devices, or the internet. A user self-reporting evaluation form and a proxy-report checklist of essential tasks was developed based on previous literature [11,34]. After completing Phase 1, the participants reported their level of confidence in using the mobile devices, and a research assistant used a standardized checklist to observe their basic skills in using the devices. At the end of Phase 4, the participants were asked to answer several questions addressing their levels of subjective confidence with, satisfaction in, and perception of usability of the mHealth training protocol (Multimedia Appendices 1 and 2).

#### Data Collection

The two local community centers from which the participants were selected were similar in terms of socio-demographic and economic factors and the proportions of older adults. The study purpose, protocol, and strategies for protection of anonymity were explained to the nurses working in the community centers. Participants were recruited through advertisements posted on bulletin boards or through word-of-mouth. Community nurses working at each center assisted with recruiting and screening study participants (eg, cognitive function and psychiatric illnesses). The data were collected using standardized self-reporting questionnaires, observation notes, and unstructured 5-minute interviews conducted between May 2016 and January 2017. To minimize bias from the community nurses, anonymous questionnaires were collected at separate places. Data collection typically took less than 30 minutes, and the participants received a small gift worth US $5 for their time and effort.

#### Data Evaluation

Based on the feasibility evaluation criteria [21], this study focused on (1) evaluating the users’ subjective appraisal of the training and study procedures and (2) identifying strategies to implement the training protocol. Therefore, both contextual and methodological considerations were identified to assist the researchers in understanding the associated barriers to be considered in future studies. Qualitative analyses of data from the structured interviews and observations were carried out, while quantitative data were reported as frequencies (%) and mean values (SD). Given the exploratory nature of this feasibility study, the statistical significance of differences between those who completed the 4-week mHealth training (n=25) and those who did not (n=2) were not tested. All statistical analyses were performed using IBM SPSS Version 23.0.

#### Ethical Considerations

The study protocol was approved by the Institutional Review Boards of College of Nursing, Yonsei University. In addition, all participants were provided with information on the voluntary nature of the study, their freedom to withdraw their enrollment, and the strategies that would be adopted to protect their anonymity and confidentiality, following which written informed consent was collected.

#### Funding

This study was supported by faculty research funds granted by the College of Nursing and the Mo-Im Kim Nursing Research Institute at Yonsei University.

## Results

### Description of Study Participants

The demographic, clinical, and technological profiles of the 27 study participants have been shown in Table 1. The mean age of the study sample was 73.33 (SD 5.98) years, and the majority of the participants were female (67%, 18/27), married or partnered (70%, 19/27), living with family members (70%, 19/27), and unemployed (96%, 26/27). Only 15% (4/27) were educated to college level or higher. The mean number of diseases exhibited by the participants was 2.07 (SD 1.21), and the mean duration of illness was 11.68 (SD 11.32) years. The majority of the participants were taking medication (70%, 19/27) to manage their chronic diseases, and approximately two thirds of the participants had previously used computers (59%, 16/27) and smartphones (67%, 18/27).

### Attrition

The attrition rates were used to evaluate the acceptability of mHealth training. The participants were classified as having completed the training if they had been through all of the phases as well as the pre- and post-evaluation. All other participants were classified as dropouts. The attrition rate for the 27 participants who took part in our mHealth training program for 4 weeks was 7% (2/27). Two dropouts (both females, less educated, widowed, living alone, diagnosed with cardiovascular diseases and exhibiting significantly depressed mood) occurred during Phase 1, and their reasons included time conflicts, emotional distress, or family discouragement.

### Assessment of Confidence and Proficiency

Phase 1 consisted of delivering group sessions, and the participant’s confidence and proficiency in using the mobile tablets were determined. The confident and proficient status indicated that the older adults could perform all eight key operating skills easily and expressed that they were “confident” or “strongly confident” without any assistance. Although participants had the opportunity to receive device training up to three times, the majority only required 1 session (88%, 22/25), and only 12% (3/25) required additional training. Twenty-five participants became proficient in completing key tasks when using the selected device and apps (mean 3.68, SD 1.75) after receiving our training (mean 3.44, SD 2.39).

**Table 1:** Characteristics of study participants.

Characteristics			Participants (N=27)
Age, mean (SD)			73.33 (5.98)
Korean version of Mini-Mental Status Examination (MMSE-K), mean (SD)			27.70 (1.73)
Korean version of Geriatric Depression Scale-Short Form (SGDS-K), mean (SD)			3.67 (3.55)
Period of living with chronic diseases, mean (SD)			11.68 (11.32)
Number of chronic diseases, mean (SD)			1.56 (0.89)
Number of comorbid conditions, mean (SD)			2.07 (1.21)
Duration of computer use (n=16, unit: month), mean (SD)			6.69 (6.18)
Duration of smartphone use (n=19, unit: month), mean (SD)			44.84 (22.49)
**Gender, n (%)**			
	Male	9 (33)	
	Female	18 (67)	
**Education, n (%)**			
	Less than middle school	9 (33)	
	Middle to high school	14 (52)	
	College or more	4 (15)	
**Marital status, n (%)**			
	Married or partnered	19 (70)	
	Widowed	8 (30)	
**Living status, n (%)**			
	Living with family members	19 (70)	
	Living alone	8 (30)	
**Employed, n (%)**			
	Employed	1 (4)	
	Unemployed	26 (96)	
**Chronic disease present (multiple answers were allowed), n (%)**			
	Cerebrovascular disease	5 (19)	
	Ischemic heart disease	4 (15)	
	Diabetes mellitus	9 (33)	
	Hypertension	14 (52)	
**Medication, n (%)**			
	Yes	19 (70)	
	No	8 (30)	
**Computer use, n (%)**			
	Past or current	16 (59)	
	Never	11 (41)	
**Smartphone use, n (%)**			
	Past or current	18 (67)	
	Never	9 (33)	
**Internet access at home, n (%)**			
	Yes	14 (52)	
	No	13 (48)	

**Table 2:** Most frequently used apps in this study.

Name of app	Provider	Major purpose	Feature participants liked most
Cardiio	Cardiio, Inc (Cambridge, USA)	Monitoring heart rate in daily life	Suggestions of exercise programs based on heart rate variability
iCare	iCare Fit Studio (Missouri, USA)	Measuring heart rate, blood pressure, vision acuity and field, lung capacity, or mood	Alarms responding to abnormal results or dramatic changes in measured data
Hypertension Protector	DONGWHA Pharm Co (Seoul, Republic of Korea)	Tracking trends in blood pressure and body weight	Personalized information based on levels of blood pressure, provided in an easy and readable manner
Diabetes Guide	Innova Think, Corp (Seoul, Republic of Korea)	Providing stepwise and diverse information on diabetes mellitus	Promotion of self-care through diet control, exercise, foot care, and infection control
Diabetes Note	Minister of Health and Welfare (Seoul, Republic of Korea)	Tracking trends in daily glucose levels	Depiction of daily, weekly, and monthly trends of blood glucose
Noom Coach	Noom, Inc (Seoul, Republic of Korea)	Facilitating lifestyle modification	Diverse types of lifestyle modification suggestions addressing sleep, stress management, prevention of depression, diet, or exercise

The mean reported confidence level for these participants, measured using a 5-point Likert-type scale where the higher scores indicated more confidence, was 3.76 (SD 1.17). The number of confident participants increased from 44% to 52% after Phase 1 training.

Men and those with higher education were more likely to learn how to use the devices and apps quickly and easily compared to women and those with lower education. Those who had used computers or mobile devices prior to this study required less training compared to the no-experience group. Previous users of Android mobile devices (72%, 18/25) required more time and information to become accustomed to Apple’s operating system.

### Users’ Satisfaction With the mHealth Device

User satisfaction was self-reported using a 5-point Likert type scale (1=*strongly disagree*, 5=*strongly agree*) for various items. Device training was considered helpful before participants initiated the mHealth training (mean 4.36, SD 1.04), and it was also found to be appropriate for health promotion (mean 4.44, SD 1.00). The device was easy to carry (mean 3.76, SD 1.27) and use (mean 3.80 SD 1.19), and the participants would recommend the device training to others (mean 4.04, SD 1.06). In addition, using the mHealth device was deemed to be less likely to interfere with their daily lives, including any concerns associated with privacy (mean 1.12, SD 0.84).

### Participants’ Preference With Regard to Selecting and Using Specific Mobile Apps

The participants requested a tailored and personalized approach toward selecting appropriate apps that took their individual health care needs into consideration from Phases 2-4. Therefore, the ratio of the number of mHealth trainers to participants became essential during Phases 3 and 4. During phases 3 and 4, one mHealth trainer assisted 2-3 participants in providing instant feedback.

Most participants preferred using interactive apps instead of those with one-way information delivery systems. For example, participants frequently used an app measuring blood pressure from the fingertip, and responded to its evaluation and instructions for lifestyle modification. In addition, they also visited websites of authorized organizations to acquire disease information; however, they very rarely revisited these pages. The favored apps among participants were those related to exercise, including tracking records, video streaming, or personalized recommendations. Table 2 summarizes the apps most frequently used by our participants. In addition, the participants also received weekly follow-up calls or text messages as reminders and reported that this contact was useful and allowed them to ask questions concerning technical problems and explore more diverse types of apps on an ‘as-needed’ basis.

## Discussion

### Principal Results and Comparison with Previous Evidence

Our protocol assisted elderly individuals participate in an mHealth training program for the self-management of chronic diseases in a community setting, where there is limited staff and resources [6,35,36]. The findings of this study revealed that acceptable feasibility of an mHealth training protocol was associated with the participants’ subjective perception, efforts to overcome barriers, and personalized approach tailored to the older adults’ characteristics [13,20]. Therefore, this study provides detailed information on the users’ experiences when implementing an mHealth training protocol among older adults. Consequently, the conceptual and methodological lessons learned are discussed to aid the development of resources and strategies for provision of mHealth training to community-dwelling older adults dealing with chronic disease management.

The emotional responses of participants at the primary stages are key factors that play a role in the successful implementation of mHealth training. Although mHealth training is considered useful, the participants that dropped out from this study or required additional mHealth training were likely to report higher depressive scores or feelings of stress. The respondents’ level of confidence is also critical for the assessment of their level of emotional distress and their ability to learn new health care training skills [10,20]. Previous studies have identified the major factors influencing successful outcomes from mHealth interventions among older populations as being independence, understanding, and confidence in accessing complex interventions [13,20]. Tracking features during periodic training and regular follow-ups are essential for the provision of positive feedback regarding progression based on objective data obtained during the mHealth training [11,35].

Furthermore, a tailored and personalized approach is necessary to ensure high levels of adherence, specifically during Phases 3 and 4 [37,38]. The mHealth trainers in this study provided health coaching rather than merely delivering information about chronic diseases [9], and the participants’ health care needs were carefully assessed before selecting apps. Strong interest in and autonomy of selection based on individual needs are a driving force facilitating any mHealth training or intervention [20]. In this study, the participants were mainly seen to utilize physiological monitoring, information-based training, and self-management app, and these findings were consistent with those of another study examining older adults in Taiwan [34]. Common features of the selected apps included recording individual data such as blood pressure and providing personal guidance and alerts based on the information provided. It appears that personalized data and timely responses enhance mHealth engagement in older adults [11,13]. Personalized information computer and technology (ICT) is one of the preconditions of achieving person-centered ICT care to manage chronic diseases [39].

In addition, a gray digital divide was seen to exist, that is, disparities using ICT device or programs in older adults. The group sessions were carefully constructed, taking into consideration the following factors: (1) mixed gender to facilitate peer learning; (2) participants exhibited similar levels of education; and (3) participants had similar previous experience of using smart devices. Adults of an advanced age required more training to become accustomed to using the device itself [10,13]. Furthermore, the mHealth trainer often had to adjust some features such as text size, brightness, and volume to overcome possible sensory and functional impairment barriers associated with the participants’ diseases or disabilities [13,20]. Gender differences, as previous studies have discussed [10,13], were identified during training and when choosing specific types of apps. There was an unintentional increase in the proportion of older and female participants in our study. Previous evidence suggests that special instructions should be provided to subgroups with low levels of literacy or e-literacy [10]. In addition, it is necessary to assess end-user’s physical conditions and preparedness prior to implementing mHealth training or intervention [40]. Moreover, clinicians should be prepared to meet all clinical and technical needs of elderly individuals receiving mHealth care. Consequently, organizational support and an interdisciplinary team approach are necessary when implementing mHealth training programs or interventions in community settings [40].

Successful self-management of chronic diseases requires empowerment and the support of informal caregivers [6] who make daily decisions regarding the patients’ health care [9]. When older adults require technical support while using smart devices and mHealth training, they are more likely to receive instant help or feedback from a significant other or a family member [38]. As demonstrated in previous studies, new devices and tasks are frequently discussed with family members, who influence the older adults’ intent to use the intervention [34,38]. Therefore, family attitudes toward telecare are a critical factor affecting the patients’ willingness to continue using the services [34]. For example, the low technical support group mentioned that their family felt pressured to learn new skills or perform additional tasks when dealing with mHealth devices or trainings [41]. Similar to older adults, family caregivers reported experiencing a greater burden when they were not fully prepared to assist the older patients with receiving mHealth care [41]. Therefore, mHealth training programs for chronic diseases must integrate goals that should be discussed with the patients and their families and health care providers [6,40]. In addition, family members should also learn how to assist older adults in using mHealth training and provide them with effective feedback [38].

Technical assistance should be provided at any phase of mHealth training. The participants of this study expressed some degree of negativity toward the use of technology in their daily practice of health care. The most common difficulties experienced by the participants were technical challenges such as unfamiliarity with using highly sensitive touch screens, the completion of technology training, or with diverse mobile apps. Many of the older adults expressed concern regarding incorrect operation of the devices or misunderstanding due to their lack of knowledge and experience [10,20,34,38]. In this study, participants received multiple device training sessions that were adjusted to their level of progress; they also received technical support for continuous use of mHealth care, as suggested by a previous study [20]. Participants were able to avail this benefit throughout the tailored and personalized approach where the technical support met their unique characteristics and needs [38].

### Implications for Current Research and Practice

This study focuses on mHealth training protocols and provides detailed information on how to develop similar training programs for older adults with chronic diseases. It is essential to ensure consistency in the initial device training to reduce any threat to internal validity [20], as limited ability to use the mHealth devices may increase the measurement error of the clinical effect [11]. Furthermore, exploring diverse retainment strategies is key in preventing dropout and providing better mHealth training [42]. Consequently, special attention should be provided to women, those who are older, and those that are not well-educated when attempting to retain older adults in mHealth training studies and practices. This study provided information about the participants’ concerns, and these should be taken into consideration when implementing evidence-based mHealth training to reduce the subjective burden [20].

The strengths of a tailored and personalized approach include provision of individualized health care services, reduction of health care expenditures, and improvement of the quality of life [43,44]. Although an mHealth care service cannot be a complete replacement for traditional face-to-face training or clinic-based treatments, it can facilitate tailored and personalized care across various settings [13]. This study provides fundamental information about mHealth training and effective delivery methods to aid clinicians caring for older Koreans living with chronic diseases. The barriers identified by this feasibility study offer opportunities for improvement to app developers, who need to consider the consumers’ perspectives of health care services.

Policymakers should consider the potential of mHealth care to meet the needs of older adults living with chronic diseases. Public service and reimbursement should be established by considering the scientific evidence [13], as mHealth training can provide diverse routes, through which health care services can be delivered to community residents [10,36]. Moreover, community centers need to disseminate mHealth technology to vulnerable populations with greater needs with regard to chronic disease management [20,36]. Future policies and public services should be equipped to take into consideration gray digital divide groups when providing mHealth services to ensure health equity.

### Study Limitations

Despite being the first feasibility study to develop an mHealth training protocol for older Korean adults, this study had several limitations. Firstly, the sample was small and had been recruited from two community sites only. Future feasibility studies should include larger sample numbers and control groups as this would enable identification of factors influencing dropouts. This, in turn, would aid clinicians in developing evidence-based strategies to enhance completion of or adherence to mHealth training [45]. Second, this study mainly used apps, websites, and video streaming, and further studies should consider expansion to other features such as text messaging, teleconferencing, and real-time feedback [10].

### Conclusions

Self-management of chronic diseases through mHealth training requires the integration of a theoretical, clinical, and technical approach, particularly when targeting older adults in a community setting. This study provided detailed information on how to develop an mHealth training protocol for self-managing chronic diseases in a community setting. The older adults exhibited a strong interest in learning how to use mHealth training devices with careful assistance from professionals. A tailored and personalized approach based on the individual characteristics and needs of the patients is necessary when implementing mHealth training protocols for older adults.

## Appendix

Multimedia Appendix 1 Confidence and proficiency in using mHealth devices.

Multimedia Appendix 2 User satisfaction with mHealth device and training: evaluation form.

## Abbreviations

eCCM: eHealth Enhanced Chronic Care Model
mHealth: mobile health
MMSE-K: Korean versions of Mini-Mental Status Examination
SGDS-K: Korean version of Geriatric Depression Scale-Short Form
