# Supplementary material for: Self-Management of Chronic Diseases Among Older Korean Adults: An mHealth Training, Protocol, and Feasibility Study
Source: JMIR Mhealth Uhealth. 2018 Jun 29;6(6):e147. doi: 10.2196/mhealth.9988 (PMC6045790; doi:10.2196/mhealth.9988)
Supplement: Multimedia Appendix 1 [file mhealth_v6i6e147_app1.pdf]

Appendix 1

Confidence and proficiency in using mHealth devices

Categories	Questionnaire	Yes	No
Question	1. Do you have confidence in using mHealth devices and related health program?		
Observation: performance of instructions in the study by participants	2.1. You can turn your mHealth device on or off.		
	2.2. You can use the touch screen.		
	2.3. You can charge the mHealth device.		
	2.4. You can control the volume of the mHealth device.		
	2.5. You can manipulate various screens.		
	2.6. You can select and install the necessary mHealth applications.		
	2.7. You can enter the information you need for your mHealth applications.		
	2.8. You can start and end the mHealth applications.		
