# Supplementary material for: Self-Management of Chronic Diseases Among Older Korean Adults: An mHealth Training, Protocol, and Feasibility Study
Source: JMIR Mhealth Uhealth. 2018 Jun 29;6(6):e147. doi: 10.2196/mhealth.9988 (PMC6045790; doi:10.2196/mhealth.9988)
Supplement: Multimedia Appendix 2 [file mhealth_v6i6e147_app2.pdf]

Appendix 2

User satisfaction with mHealth Device and training: evaluation form

Please think about your use of the mHealth device and mark whether you agree or disagree with the following statements. Check only one box on each line and answer all questions.

	**Strongly disagree**	**Disagree**	**Neutral**	**Agree**	**Strongly agree**
1. Overall, the mHealth device was:					
easy to use.					
easy to track my health status.					
easy to carry.					
2. The training in how to use the mHealth device prior to using the device was helpful.					
3. Using the mHealth device intruded on my privacy.					
4. I felt confident in using the mHealth device.					
5. I would recommend this mHealth device and the selected applications to others.					
